# Molecular Characterization and Defense Functions of the Nile Tilapia (*Oreochromis niloticus*) *DnaJ B9b* and *DnaJ C3a* Genes in Response to Pathogenic Bacteria under High-Temperature Stress Conditions

**DOI:** 10.3390/biom11101509

**Published:** 2021-10-13

**Authors:** Prapansak Srisapoome, Kubpaphas Thummabancha, Ratree Wongpanya

**Affiliations:** 1 Laboratory of Aquatic Animal Health Management, Department of Aquaculture, Faculty of Fisheries, Kasetsart University, Chatuchak, Bangkok 10900, Thailand; t.kubpaphas@gmail.com; 2 Center of Advanced Studies for Agriculture and Food, Kasetsart University Institute for Advanced Studies, Kasetsart University, Bangkok 10900, Thailand; 3 Center of Excellence in Aquatic Animal Health Management, Faculty of Fisheries, Kasetsart University, Chatuchak, Bangkok 10900, Thailand; 4 Department of Biochemistry, Faculty of Science, Kasetsart University, Bangkok 10900, Thailand; fscirtw@ku.ac.th

**Keywords:** molecular chaperone, heat shock protein, Hsp40, aquaculture, *Streptococcosis*, columnaris, RNAi

## Abstract

DnaJ proteins or heat shock protein 40s (HSP40s) form one of the largest heat shock protein families. In this study, 2 cDNAs encoding Nile tilapia (*Oreochromis niloticus*) DnaJ proteins (On-DnaJ B9b and On-DnaJ C3a) were successfully cloned and characterized. The structures and organizations of these two genes are first reported in the present study. *On-DnaJ B9b* is approximately 2.1 kb long and contains 2 exons and 1 intron, while *On-DnaJ C3a* is approximately 12 kb long and contains 12 exons and 11 introns. Under normal conditions, *On-DnaJ B9b* mRNA is highly expressed in gonad and trunk kidney tissues, while *On-DnaJ C3a* transcripts are abundantly expressed in gills, intestine, liver, and trunk kidney tissues. Following pathogenic infections, the expression of both genes is induced in the liver, spleen and head kidney tissues of Nile tilapia that were infected with two virulent pathogenic bacteria, *Streptococcus agalactiae* and *Flavobacterium columnare*. Silencing of these two genes was first carried out, and the results clearly indicated their crucial roles under both heat and bacterial stress conditions. The fundamental knowledge obtained from this study indicates the characteristic basic biofunctions of heat shock proteins in the regulation of intracellular proteins during infection, which involve preventing protein aggregation, promoting protein refolding, and activating unfolded protein degradation.

## 1. Introduction

Nile tilapia (*Oreochromis niloticus*) is a tropical teleost fish that is cultured worldwide because of its substantial economic and trade value and its high annual production rate [1]. Since it is classified as a fast-growing, eurythermal and euryhaline aquatic animal, it was introduced for cultivation in many areas around the world [1]. As such, its culture has increased and become extensive. However, poor management of this culture or rapid changes in water quality have resulted in the presence of undesirable pathogenic microorganisms, which can negatively affect the health of these fish, leading to their death and the loss of their economic trade value [2]. An increase in high temperature (>31 °C) or rapid fluctuation in this factor were observed to induce high susceptibility to some bacterial diseases, such as streptococcosis and columnaris [3]

Streptococcosis is a common infectious bacterial disease of tilapia worldwide caused by *Streptococcus* spp. The most common species that usually causes streptococcosis outbreaks is *Streptococcus agalactiae*. This bacterium is gram-positive and exhibits a spherical shape, a red blood cell lysing capability, lactic acid production during the fermentative process and catalase negativity [3]. Diseased fish display septicemia and severe meningoencephalitis [4]. Generally, infected fish exhibit skin hemorrhage, exophthalmia, ascites, uncontrollable swimming and anorexia [5].

Columnaris disease is a bacterial infection that has a high mortality rate in warm-water fish [6]. This disease is characterized by gill necrosis, grayish-white patches on the body, skin erosion, and fin rot [7]. The main causative agent of this disease is *Flavobacterium columnare*, which is a gram-negative bacterium with a long rod shape and gliding motility [8]. *F. columnare* is a fastidious microorganism that needs to be grown in specific media, such as Shieh’s selective medium [9]. The rapid expansion of these diseases throughout tilapia farms has become a major concern to researchers who are working to improve prophylactic strategies.

Many scientific papers have reported that during infection and inflammatory states, various heat shock proteins (HSPs) show significantly upregulated expression compared with those under normal conditions [10,11,12,13,14]. Generally, HSPs can be classified into many subclasses depending on their molecular weights [11]. The main functions of HSPs involve the folding of native proteins, the refolding of denatured proteins, the degrading of misfolded proteins and acting as secondary signal messengers in cells [15]. HSPs are widely distributed throughout cellular compartments including the nucleus, cytoplasm, endoplasmic reticulum (ER) and Golgi apparatus [16]. HSPs also play a major role in intracellular antigenic processing in antigen presenting cells (APCs) through major histocompatibility complex (MHC) class I [10,17].

Recently, an increasing number of relevant studies have established a correlation between ER stress and infections [18,19,20]. The unfolded protein response (UPR) is a cellular stress process that occurs in the ER lumen [21]. *DnaJ* (*HSP40*), *ER DnaJ* (*ERdj*) homolog and *microvascular differentiation gene 1* (*Mdg1*) are a group of cellular proteins located in the ER lumen and are rapidly expressed in response to UPR induction to regulate and maintain proper folding of denatured proteins in the lumen [22,23]. Under cellular stress conditions, DnaJ proteins trap unfolded/misfolded proteins. These molecules carry impaired proteins to bind immunoglobulin protein (BiP) or 78 kDa glucose-regulated protein (GRP78), which is strictly located in the ER lumen and belong to the HSP70 family [24,25]. BiP later initiates binding and refolds client proteins into their correct structure so that they can function properly. Recently, the DnaJ B9b and DnaJ C3a proteins were found to be induced during ER stress [25,26]. DnaJ B9b is involved in the ER-associated degradation (ERAD) pathway; it plays a major role in the degradation of ERAD substrates by sending clients through the integral membrane protein Derlin-1, which is an ERAD component, to promote proteasomal degradation [25,26,27]. DnaJ C3a binds to unfolded substrates via its tetratricopeptide repeat (TPR) motif, which maintains protein folding homeostasis and promotes protein refolding in the ER lumen under UPR activation [25,28,29].

The complementary DNAs (cDNAs) that encode the DnaJ proteins important for UPR and ER stress responses, the structures of the encoding genes, and the biological defense functions of these proteins in Nile tilapia have not been characterized. Therefore, this study was conducted to better define the chaperone requirements and elucidate the crucial biological functions of DnaJ B9b and DnaJ C3a under various conditions in the target organism. The results of this study provide crucial information regarding the full-length cDNAs that encode the Nile tilapia *DnaJ subfamily B member 9b* (*On-DnaJ B9b*) and *subfamily C member 3a* (*On-DnaJ C3a*) genes. The gene organization of these genes was demonstrated. Additionally, we evaluated *On-DnaJ B9b* and *On-DnaJ C3a* gene expression levels in Nile tilapia in response to infectious states, especially following *S. agalactiae* and *F. columnare* infections, which cause severe issues in Nile tilapia aquaculture worldwide. Biological function analyses of these two genes were performed via gene knockdown techniques. These findings may provide important information at the molecular level for understanding host-pathogen interactions and cellular stress responses in bacteria-infected Nile tilapia.

## 2. Materials and Methods

### 2.1. Experimental Animals

Juvenile Nile tilapia weighing approximately 30 g were purchased from a Nile tilapia farm in central Thailand. The fish were acclimatized in a 1000-l fiberglass tank filled with dechlorinated tap water at the Department of Aquaculture, Faculty of Fisheries, Kasetsart University. The tilapia were fed twice daily with commercial pellet feed. Twenty percent of the water was changed every two days, and feces and waste were removed twice a day. The Nile tilapia were acclimatized under laboratory conditions for 7 days prior to the start of the experiment. The water was continuously aerated using an air stone and oxygen generator.

### 2.2. Cloning and Characterization of Nile Tilapia cDNA Encoding the On-DnaJ B9b and On-DnaJ C3a Genes

The head kidney and spleen of healthy adult Nile tilapia were dissected and stored in TRIzol reagent (Gibco BRL, Waltham, MA, USA) for total RNA extraction. Total RNA was extracted according to the manufacturer’s instructions. For the extraction of total RNA, all processes were conducted using a FastPrep^®^ homogenizer (MP Biomedicals, Santa Ana, CA, USA) as recommended by the manufacturer’s protocol. Only mRNA was isolated using a QuickPrep Micro mRNA Purification Kit (Amersham Biosciences, Piscataway, NJ, USA) according to the manufacturer’s instructions. A 500 µg mRNA sample was obtained from each organ, and then, all the samples were pooled together. The ready-to-use 5’ and 3’ cDNA templates for RACE-PCR were synthesized as recommended by the manufacturer’s protocol (Clontech, Fitchburg, WI, USA) by using 1 µg/µL of pooled mRNA. Specific primer pairs for On-DnaJ B9b F/On-DnaJ C3a F and On-DnaJ B9b R/On-DnaJ C3a R (Table 1) were generated to conduct 3’ and 5’ RACE-PCR. The specific primers used in this study were designed from a partial EST clone cDNA from a Nile tilapia cDNA library (GenBank accession number FF281612 for *On-DnaJ B9b* and FF280511 for *On-DnaJ C3a*), which was used in 5’ and 3’ RACE-PCR to recover missing transcript information from both the 5’ and 3’ directions of the *On-DnaJ B9b* and *On-DnaJ C3a* cDNAs. The thermal cycler reaction conditions for the 5’ and 3’ RACE-PCR were as follows: 5 cycles of 94 °C for 30 s and 72 °C for 3 min; followed by 5 cycles of 94 °C for 30 s, 70 °C for 30 s, 72 °C for 3 min; and 25 cycles of 94 °C for 30 s, 68 °C for 30 s, and 72 °C for 3 min. The 5’ and 3’ RACE-PCR products were separated by gel electrophoresis using a 1% agarose gel to determine the amplicon sizes. The products were purified from the gel using a HiYield^TM^ Gel/PCR DNA fragment extraction kit (RBC Bioscience, New Taipei City, Taiwan).

The purified products were then ligated into the pGEM^®^-T Easy vector (Promega, Madison, WI, USA), and the recombinant plasmids were cloned into competent *Escherichia coli* DH5 alpha cells [30]. The plasmids containing inserts of interest were then sequenced using a Thermo Sequenase fluorescently labeled primer cycle sequencing kit (Amersham Pharmacia Biotech, Piscataway, NJ, USA) with the M13 forward and reverse primers (Macrogen, Inc., Geumcheon-gu, Seoul, Korea).

The homology of the nucleotide sequences was subsequently tested against sequences available in the GenBank database using both the BlastX and BlastN programs (http://www.ncbi.nlm.nih.gov/ (accessed on 9 March 2021)). The nucleotide sequences of both the 5’ and 3’ directions were aligned with each other, and the joining or overlapping regions were consequently represented in the full-length cDNAs of both the *On-DnaJ B9b* and *On-DnaJ C3a* genes. The hydrophobic leader sequence was confirmed using SignalP 3.0 (http://www.cbs.dtu.dk/services/SignalP (accessed on 9 March 2021)). The theoretical isoelectric point (p*I*) and molecular weight of On-DnaJ B9b and On-DnaJ C3a were determined using ExPASy (http://www.expasy.org/ (accessed on 9 March 2021)). The similarity and identity of the nucleotide and amino acid sequences of the *On-DnaJ B9b* and *On-DnaJ C3a* genes from Nile tilapia and other organisms were computed using the MatGat program, version 2.02 (http://bitincka.com/ledion/matgat (accessed on 9 March 2021)). To predict the three-dimensional (3D) structures of On-DnaJB9b and On-DnaJC3a proteins, homology modeling was carried out using the SWISS-MODEL database (https://www.swissmodel.expasy.org (accessed on 9 March 2021)). The predicted model structures were then visualized with PyMOL software (Schrödinger, Inc., New York, NY, USA).

### 2.3. Gene Organization Structural Analysis of the Nile Tilapia DnaJ B9b and DnaJ C3a Genes

Next, the full-length cDNAs of the Nile tilapia *DnaJ B9b* and *DnaJ C3a* genes obtained from the above experiments were blasted against sequence information available in the Ensembl databases (www.ensembl.org (accessed on 29 April 2021)). The exon and intron information for these two genes was evaluated and compared with the *DnaJ B9b* and *DnaJ C3a* genomic organizations from all other organisms in the available databases.

### 2.4. Evolutionary Tree Construction of the DnaJ B9b and DnaJ C3a Genes

The full-length cDNAs of the Nile tilapia *DnaJ B9b* and *DnaJ C3a* genes were excluded from the 5’ and 3’ untranslated region (UTR) sequences to obtain only the open reading frame (ORF) sequences, which were translated to determine the amino acid information. The other known *DnaJ B9b* and *DnaJ C3a* genes from other vertebrates, including fish, amphibians, birds and mammals, which are available in the GenBank database (http://www.ncbi.nlm.nih.gov/ (accessed on 29 April 2021)), were carefully examined. All sequences were aligned using ClustalW (http://www.clustal.org (accessed on 29 April 2021)). A phylogenetic tree was ultimately constructed with the UPGMA method through the MEGA 5.05 program (http://www.megasoftware.net (accessed on 29 April 2021). Bootstrapping with 1000 bootstrap replications was utilized to assess the reliability of the obtained tree. The Nile tilapia *HSP70* gene (accession no. ACJ03597), which was evolutionarily distant from these two heat shock proteins, was used as the outgroup.

### 2.5. Normal Tissue Distribution of On-DnaJ B9b and On-DnaJ C3a Gene Expression in Healthy Nile Ti-Lapia

To examine the expression patterns of the *On-DnaJ B9b* and *On-DnaJ C3a* genes in various healthy Nile tilapia organs (approximately 30 g), we collected thirteen organs: the brain, gills, gonads, heart, anterior kidneys, intestine, liver, muscle, peripheral blood leukocytes (PBLs), skin, spleen, stomach and posterior kidneys. Total RNA was extracted from all tissues using TRIzol reagent, as described in Section 2.2. The quality and quantity of the total RNA were measured using a NanoDrop spectrophotometer (NanoDrop 2000, Thermo Scientific, Waltham, MA, USA). The final concentration was adjusted to 1 µg of total RNA, and a RevertAid First Strand cDNA Synthesis Kit (Fermentas, Waltham, MA, USA) was used to synthesize the first-strand cDNA. Real-time RT-PCR was performed in triplicate for each organ using 1 µg of first-strand cDNA. For analysis of the *On-DnaJ B9b* and *On-DnaJ C3a* mRNA levels in each organ, 4.75 µL of distilled water, 6.25 µL of Brilliant^®^ II SYBR^®^ Green qPCR Master Mix (Stratagene, La Jolla, CA, USA) and 1 µL of 10 mM specific primers for On-DnaJ B9b F/On-DnaJB 9b R and On-DnaJ C3a F/On-DnaJ C3a R (Table 1) were used. Beta-actin gene expression, with specific primers for On-Beta-actin F/On-Beta-actin R (Table 1), was used as a reference gene. The real-time PCR conditions were as follows: an initial denaturation step at 95 °C for 10 min, followed by 40 cycles of 95 °C for 30 s, 55 °C for 30 s, and 72 °C for 1 min, and a final cycle of 95 °C for 1 min, 55 °C for 30 s, and 95 °C for 30 s. The threshold cycle (CT) value was recorded for further calculations used to analyze the expression patterns of the *On-DnaJ B9b* and *On-DnaJ C3a* genes in each organ. The relative expression ratios were normalized to the expression levels of each gene in the brain using the 2^−∆∆CT^ method [31].

### 2.6. Quantitative Real-Time RT-PCR (qRT-PCR) Analyses of the On-DnaJ B9b and On-DnaJ C3a Genes in Response to Systemic Pathogenic Infection

#### 2.6.1. Preparation of Bacterial Suspension

The two pathogenic bacteria, *S. agalactiae* and *F. columnare*, were isolated from infected fish courtesy of the Laboratory of Aquatic Animal Health Management, Department of Aquaculture, Faculty of Fisheries, Kasetsart University, Thailand. *S. agalactiae* was enriched in tryptic soy broth (TSB) and incubated at 32 °C for 18 h. The bacterial cells were centrifuged at 600× *g* for 15 min and washed twice with 0.85% NaCl. The bacterial suspension concentration was adjusted with a spectrophotometer (Spectronic BioMate 3, Thermo Fisher Scientific, Waltham, MA, USA) at a specific wavelength of 600 nm with an optical density (OD) of 0.6, which was equal to 1 × 10^9^ colony forming units per milliliter (CFU/mL). The suspension was diluted 100-fold with normal saline to obtain a bacterial concentration of 1 × 10^7^ CFU/mL. *F. columnare* enrichment was also performed, and the cells were cultured in Shieh’s broth and incubated at 32 °C for 18 h. The bacterial cells were precipitated and washed with normal saline as described above. The bacterial concentration (1 × 10^9^ CFU/mL) was spectrophotometrically adjusted to an OD of 2.1 with a specific wavelength of 525 nm. A 1 × 10^7^ CFU/mL bacterial suspension was obtained with the dilution method described above.

#### 2.6.2. Experimental Animal Design

One hundred fifty healthy Nile tilapia (approximately 30 g weight) were acclimatized for seven days before the start of the experiment. The fish were randomly separated into five groups (30 fish each) and reared in experimental blue tanks containing 150 L of tap water. The rearing water was continuously aerated using air stones and an oxygen generator. During the experimental period, excretion and feces were removed daily, and 20% of the water was changed every two days. After acclimatization, all fish in group 1 were intraperitoneally injected with 0.1 mL of normal saline solution (0.85% NaCl), which was given as a control treatment. The animals in groups 2 and 3 were intraperitoneally injected with 0.1 mL of *S. agalactiae* at concentrations of 1 × 10^7^ and 1 × 10^9^ CFU/mL, respectively. The animals in groups 4 and 5 were injected with 0.1 mL of *F. columnare* at concentrations of 1 × 10^7^ and 1 × 10^9^ CFU/mL, respectively, via the same route of administration as the previous groups. After injection, the liver, spleen and head kidney, which are three vital organs that are involved in the immune response, were harvested from three fish in each group and dissected at 6 h, 12 h, 1 day, 2 days, 3 days and 7 days after pathogenic administration. These samples were stored in TRIzol for total RNA extraction.

#### 2.6.3. qRT-PCR Analysis

Total RNA from each group at the designed time points was extracted and converted to first-stand cDNA following the manufacturer’s protocol. One microgram of first-strand cDNA of each organ at different time points was separately used as the target template for qRT-PCR analysis, which was conducted to evaluate the *On-DnaJ B9b* and *On-DnaJ C3a* gene expression levels with a method similar to that described above. The relative expression ratios of each gene were normalized with *β-actin* gene expression levels with the 2^−∆∆CT^ method [31].

### 2.7. Silencing Analysis and Effects of On-DnaJ B9b and On-DnaJ C3a Gene Knockdown under Normal Conditions

#### 2.7.1. Experimental Design and Double-Stranded RNA (dsRNA) Preparation

Five hundred Nile tilapia fingerlings (approximately 5 g) were acclimatized under laboratory conditions with the methods described above. Fifteen fish each were randomly moved and maintained in 4 different 100-l glass tanks for seven days. In this study, the water temperature was set at 28 ± 1.5 °C using a controlled heating system (HOPAR^TM^ K-339, Chosion, China). During this time, the gene-specific primers DnaJB9bT7_F/DnaJB9bT7_R and DnaJC3aT7_F/DnaJC3aT7_R (Table 1) were designed to obtain DNA templates containing a T7 promoter. These primers were used to amplify cDNA from Section 2.2 with the same protocol, and the targeted PCR fragments were cloned into the pGEM T-easy vector with the above process. The specific dsRNA sequences for *On-DnaJ B9b* (ds*On*-*DnaJ B9b*) and *On-DnaJ C3a* (ds*On-DnaJ C3a*) and the green fluorescent protein (ds*GFP*) gene were synthesized using the T7 RiboMAX™ Express RNAi System (Promega Corporation, Madison, WI, USA) and purified following a previous method described by the manufacturer’s protocol.

#### 2.7.2. dsRNA Delivery and qRT-PCR Analysis for Gene Knockdown of the *On-DnaJ B9b* and *On-DnaJ C3a* Genes

All fish prepared in 4 tanks in Section 2.7.1. were intraperitoneally injected with different conditions as follows: in the first to fourth tanks, fish were injected with 50 μL of phosphate-buffered saline (PBS, pH 7.4), PBS+5 μg dsOn-DnaJ B9b, PBS+5 μg dsOn-DnaJ C3a and PBS+5 μg dsGFP. After injection, at 6, 12, 24, 48, 72, 96 and 120 h, gill and liver samples of three fish in each group were collected. Total RNA was extracted, and first-strand cDNA synthesis was performed with the methods described above. qRT-PCR analyses of the *On-DnaJ B9b* and *On-DnaJ C3a* genes at each time point were conducted with the same protocol described in Section 2.5.

### 2.8. Silencing Analysis and Effects of On-DnaJ B9b and On-DnaJ C3a Gene Knockdown at High Water Temperature

Fifteen fish each in Section 2.7.1. were randomly moved and maintained in five different 100-l glass tanks under stable water temperature at 28 ± 1.5 °C with the same method described above for seven days. Prior to dsRNA induction, the water temperature in the second to fifth tanks was gradually increased to 35 ± 2.1 °C within 1 h. Then, all fish prepared in five tanks were intraperitoneally injected with different conditions as follows: in the first to fifth tanks, fish were injected with 50 μL of PBS (pH 7.4), PBS, PBS+dsGFP, PBS+5 μg dsOn-DnaJ B9b, and PBS+5 μg dsOn-DnaJ C3a. During this period, the behavior and mortality of fish in each group were recorded until day seven.

### 2.9. Effects of On-DnaJ B9b and On-DnaJ C3a Gene Silencing under High-Temperature Stress with S. agalactiae Infection

Ten fish each prepared in Section 2.7. were randomly placed in 15 different 100-l glass tanks for seven days, and then the water temperature of the tanks was raised to 35 ± 2.1 °C as described above. Then, every fish in tanks 1–3 and 4–6 was first injected with PBS (pH 7.4). The fish in tanks 7–9, 10–12 and 13–15 were intraperitoneally injected with 50 μL of PBS+5 μg dsGFP, PBS+5 μg dsOn-DnaJ B9b and PBS + 5 μg dsRNA of On-DnaJ C3a, respectively. In the next 2 h, fish in tanks 4–15 were injected with 50 μL of 1 × 10^9^ CFU/mL of *S. agalactiae* suspension. After injection, fish in each group were maintained in their tanks. Behavior and mortality were recorded every 6 h during the first two days and daily until day seven.

### 2.10. Statistical Analysis

The relative expression ratios of the *On-DnaJ B9b* and *On-DnaJ C3a* genes in each organ in Section 2.5 and each organ at different time points in each group in Section 2.6 and Section 2.7 and accumulative mortality in Section 2.8 and Section 2.9. were statistically determined using one-way analysis of variance (ANOVA) followed by Duncan’s new multiple range test (DMTR) to evaluate the mean differences in each organ or treatment with 95% confidence intervals (*p* < 0.05).

## 3. Results

### 3.1. Structural Characterization of the Nile Tilapia cDNAs Encoding the On-DnaJ B9b and On-DnaJ C3a Genes

**Figure 1:**
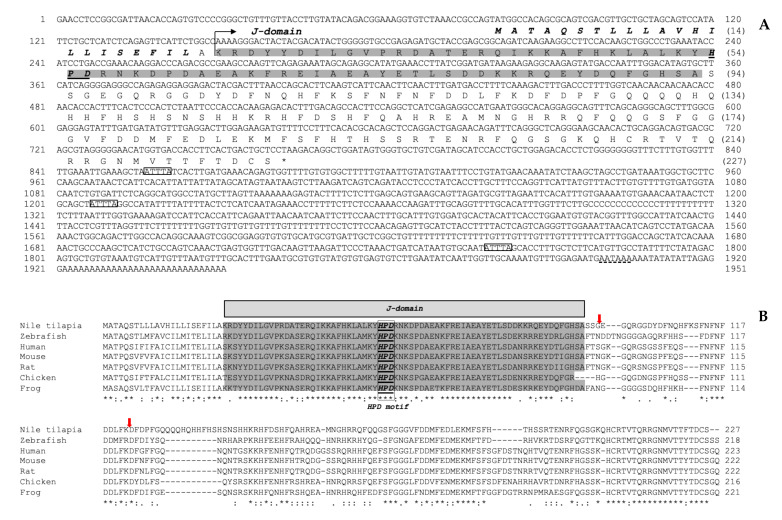
Full-length cDNA encoding the Nile tilapia *DnaJ B9b* gene (**A**). The upper and lower lines indicate the nucleotide and amino acid sequences, respectively. The leader sequence is in bold and italics. The J domain is highlighted, and the conserved HPD motif is bolded and underlined. The instability motifs (ATTTA) are shown in boxes. The polyadenylation signal (AATAAA) is underlined with a dashed line. Amino acid sequence alignments of Nile tilapia, zebrafish, human, mouse, rat, chicken and frog *DnaJ B9b* genes (**B**). Two red arrows indicate G/F region. The asterisks (*) indicate conserved residues in all sequences. Colons (:) and dots (.) indicate the high and low conserved residues in most sequences, respectively. The conserved J domain is highlighted in gray. The HPD motif is bolded and underlined in the box.

The total length of the *On-DnaJ B9b* cDNA was 1951 bp, with an 80 bp 5’UTR, a 681 bp coding region equal to 227 amino acid residues, and a 1187 bp 3’UTR. Furthermore, a hydrophobic leader sequence was found at amino acid positions 1 to 22, and three instability motif positions were found in the 3’UTR (Figure 1A). The calculated theoretical p*I* and molecular weight of *On-DnaJ B9b* were 6.83 and 26.58 kDa, respectively. The multisequence alignment results between *DnaJ B9b* cDNA of Nile tilapia and other known organisms revealed that this sequence has a signal peptide (22 aa). Additionally, *On-DnaJ B9b* cDNA has a J domain that contains a conserved His-Pro-Asp (HPD) motif, which was predicted to be a BiP-ATPase binding protein (Figure 1B). The g/F region was found downstream of the J domain. No N-linked glycosylation sites (NXS or NXT) were observed throughout the sequence. A ten-amino acid QH-rich insertion was only observed in *On-DnaJ B9b*. Additionally, the Nile tilapia and zebrafish *DnaJ B9b* cDNAs had six amino acids that were detected near the C-terminal end (Figure 1B). Based on information from this characterization, the lack of zinc-finger motifs and the organization of the J domain-G/F region-peptide binding fragment pattern, On-DnaJ B9b belongs to type II Hsp40.

**Figure 2:**
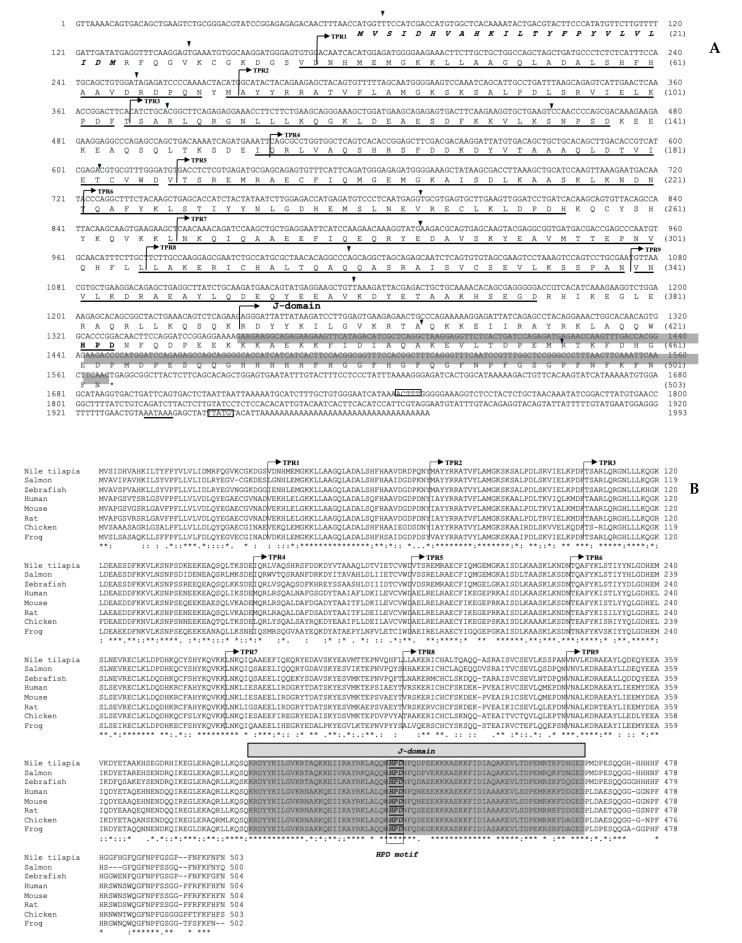
Full-length cDNA encoding the Nile tilapia *DnaJ C3a* gene (**A**). The upper and lower lines indicate the nucleotide and amino acid sequences, respectively. The leader sequence is in shown in bold and italics. TPRs no. 1 to 9 are underlined. The J domain is highlighted, and the conserved HPD motif is bolded and underlined. The instability motifs (ATTTA) are shown in boxes. The polyadenylation signal (AATAAA) is underlined with a dashed line. Amino acid sequence alignments of Nile tilapia, salmon, zebrafish, human, mouse, rat, chicken and frog *DnaJ C3a* genes (**B**). The asterisks (*) indicate conserved residues in all sequences. Colons (:) and dots (.) indicate high and low conserved residues in most sequences, respectively. The conserved J domain is highlighted in gray. The HPD motif is bolded and underlined in the box.

The completely characterized full-length cDNA encoding the Nile tilapia *DnaJ C3a* gene is 1993 bp in total length, with a 58 bp 5’UTR, a 1509 bp ORF that corresponds to 503 aa, and a 423 bp 3’UTR (Figure 2A). The calculated pI and molecular weight of *On-DnaJ C3a* were 7.09 and 57.79 kDa, respectively. The multisequence alignments of the Nile tilapia *DnaJ C3a* cDNA compared with those of other organisms indicated that the *On-DnaJ C3a* cDNA contains a 24 aa leader sequence, 9 TPR domains, a J domain with an HPD motif, and 2 instability sequences. This organization causes *On-DnaJ C3a* to cluster into type III Hsp40 (Figure 2B).

A comparison of the *On-DnaJ B9b* gene with other known *DnaJ B9b* genes from higher vertebrates showed that their nucleotide identity scores were 60.9–64.6%, and their amino acid identity and similarity scores were 60.9–64.7% and 77.1–81.1%, respectively. The On-DnaJ B9b gene had strong nucleotide identities of 77.7% and 77.3% with those of pufferfish and Japanese medaka, respectively, and its amino acid identity scores were 80.9% and 77.7%, indicating close relationships with pufferfish and Japanese medaka, respectively (Supplemental material Table S1).

A comparison of the *On-DnaJ C3a* gene with other known *DnaJ C3a* genes from higher vertebrates showed that their nucleotide identity scores were 66.0–68.0%, and their amino acid identity and similarity scores were 67.5–69.8% and 82.7–85.9%, respectively. Additionally, the Nile tilapia *On-DnaJ C3a* gene had 85.2% and 88.5% nucleotide and amino acid sequence identities, respectively, and exhibited 95.0% amino acid similarity with that of Japanese medaka (Supplemental material Table S2).

**Figure 3:**
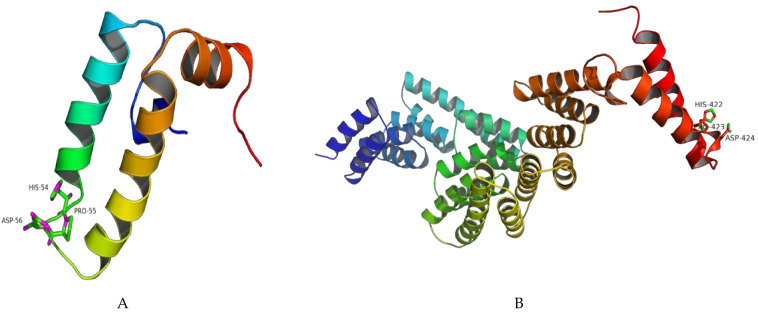
Model of the On-DnaJB9b (**A**) and On-DnaJC3a (**B**) homology predicted by the SWISS-MODEL program. The HPD motifs are indicated by numbering the amino acid residue. The color of the model is set as a rainbow where the N-terminus is colored blue, the C-terminus is colored red, and residues between 2 termini are rainbow color gradients.

Homology modeling revealed that *On-DnaJB9b* exhibited the greatest similarity to DnaJ subfamily B member 9 in humans (PDB 2ctr.1. A), with an identity of 77.46% (Figure 3A and Supplemental material Figure S1). The conserved His-Pro-Asp (HPD) motif of *On-DnaJ B9b* was found in a random coil region. The *On-DnaJC3a* model similarly showed that *On-DnaJC3a* exhibited the highest similarity to DnaJ homolog subfamily C member 3 in humans, the cochaperone P58 (IPK) (PDB 2y4t.3. A), with an identity of 72.37%. The model structure of *On-DnaJC3a* was found to contain an HPD motif situated next to the loop between the last two alpha-helices (Figure 3B and Supplemental Material Figure S2).

### 3.2. Organization of the On-DnaJ B9b and On-DnaJ C3a Genes

**Figure 4:**
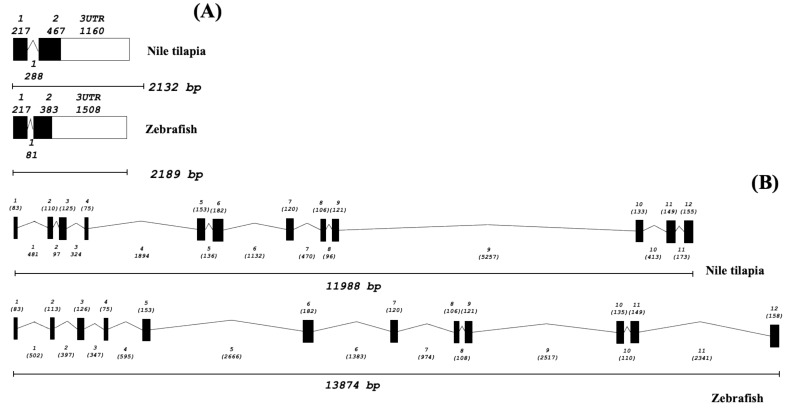
Genomic organization of the Nile tilapia *DnaJ B9b* (**A**) and *DnaJ* C3a (**B**) genes. The numbers above each gene structure indicate the exon numbers and their lengths in bp. The numbers below each gene structure indicate the intron numbers and their lengths in bp.

A comparative analysis of the full-length *On-DnaJ B9b* and *On-DnaJ C3a* cDNAs and genomic DNA sequences that are available in the Ensembl databases demonstrated that the *On-DnaJ B9b* gene has a short, fully functional structure that includes 2132 bp organized into two exons and one intron. The first exon (217 bp) contains a 288-bp intron. The last exon (267 bp) is linked to a 1160-bp 3’UTR. The *On-DnaJ C3a* gene, which comprises 11,988 bp, is organized into 12 exons and 11 introns. Its exon lengths are 75–182 bp, while its intron lengths are 96–5257 bp (Figure 4).

### 3.3. Evolutionary Analysis of the On-DnaJ B9b and On-DnaJ C3a Genes

The *On-DnaJ B9b* and *On-DnaJ C3a* gene phylogenetic trees revealed that these two genes were obviously divided into two individual groups. In each evolutionary clade, the tree was obviously split into two major clusters, which included higher and lower vertebrates. For the *DnaJ B9b* gene, zebrafish (*Danio rerio*) *DnaJ B9b* was grouped into the higher vertebrate branch. The *On-DnaJ B9b* gene was classified into the Osteichthyes (bony fish) group, which is highly related to pufferfish (*Takifugu rubripes*) (Class Actinopterygii, Order Tetraodontiformes) and Japanese medaka (*Oryzias latipes*) (Class Actinopterygii, Order Beloniformes). The *On-DnaJ C3a* gene was also grouped into the Osteichthyes branch and was closely related to Japanese medaka (Supplemental material Figure S3).

### 3.4. On-DnaJ B9b and On-DnaJ C3a Gene Tissue Distribution in Healthy Nile Tilapia

**Figure 5:**
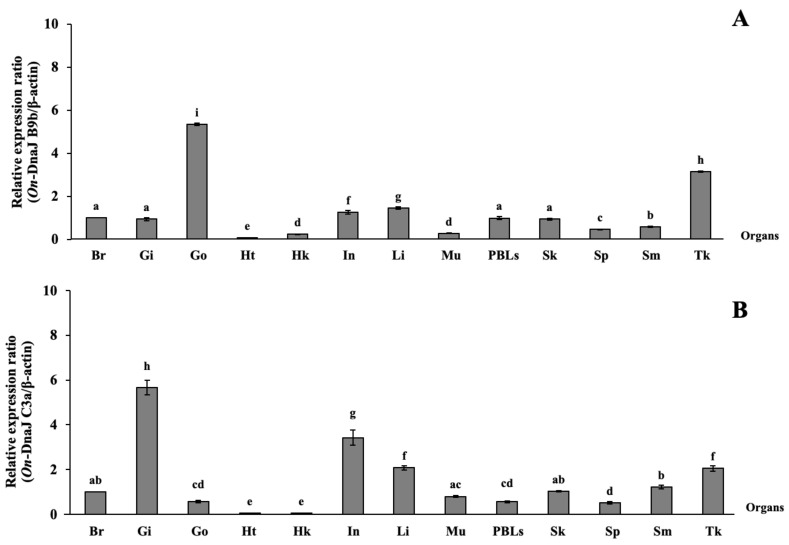
*On-DnaJ B9b* (**A**) and *DnaJ C3a* (**B**) mRNA expression in normal tissues. Each bar indicates the expression levels in each organ. The different letters above each bar indicate a significant difference. *p* < 0.05 was considered significant (Br = brain, Gi = gills, Go = gonad, Ht = heart, Hk = head kidney, In = intestine, Li = liver, Mu = muscle, PBLs = peripheral blood leukocytes, Sk = skin, Sp = spleen, Sm = stomach, Tk = trunk kidney).

qRT-PCR analyses of the *On-DnaJ B9b* and *On-DnaJ C3a* genes in various healthy Nile tilapia tissues indicated that the *On-DnaJ B9b* transcript was significantly expressed in gonad and trunk kidney tissues and that its expression levels were 5.35 ± 0.05- and 3.15 ± 0.02-fold higher than the expression levels in the brain, which was used as a tissue calibrator. In contrast, its expression was 0.08 ± 0.01-fold higher in the heart, 0.24 ± 0.02-fold higher in the head kidneys and 0.28 ± 0.01-fold higher in muscle tissue than in the brain (Figure 5A). *On-DnaJ C3a* mRNA was highly expressed in the gills (5.66 ± 0.33-fold), intestine (3.42 ± 0.34-fold), liver (2.08 ± 0.10-fold) and trunk kidneys (2.05 ± 0.12-fold); however, its expression was lower in the heart (0.05 ± 0.01-fold) and head kidneys than in the brain (Figure 5B).

### 3.5. On-DnaJ B9b and On-DnaJ C3a Gene Expression Levels in Nile Tilapia Infected with Two Pathogenic Bacteria

**Figure 6:**
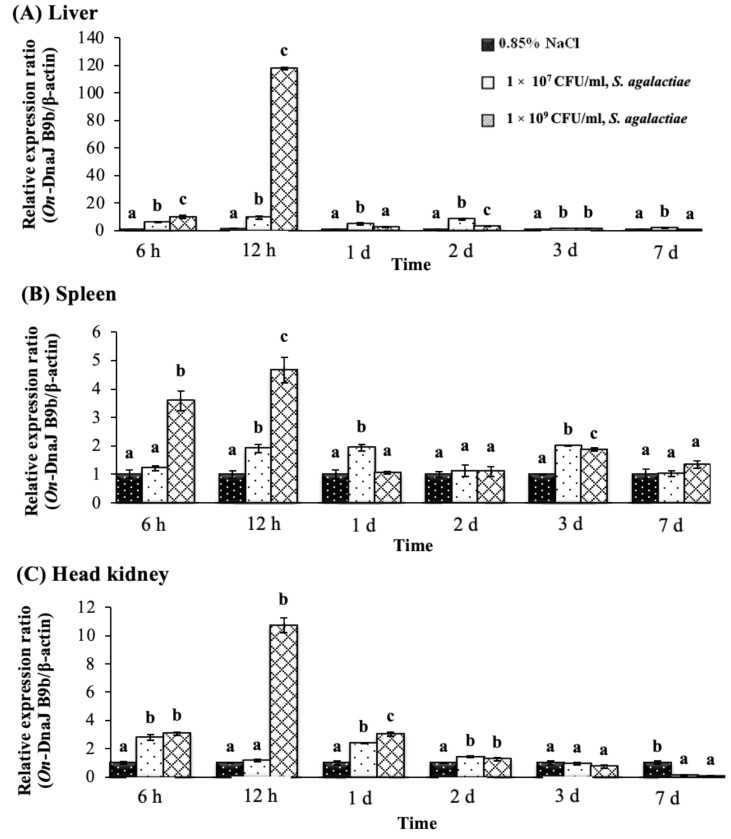
Nile tilapia *DnaJ B9b* transcript levels in fish exposed to *S. agalactiae* at 1 × 10^7^ and 1 × 10^9^ CFU/mL at different time points in the liver (**A**), spleen (**B**) and head kidney (**C**). The different letters on each bar indicate significant differences (*p* < 0.05). This scheme is also used for Figure 7, Figure 8, Figure 9, Figure 10 and Figure 11.

During the experimental periods, there were no deaths of the fish in either bacterial challenge group, but some fish showed significant lethargy in 48–96 h. The results showed that after fish were injected with *S. agalactiae*, the *On-DnaJ B9b* transcript levels in the liver were significantly upregulated in all treatment groups compared with the control group in a dose-dependent manner, especially at early time points following infection (6–12 h). The highest upregulation of gene expression (117.79 ± 1.15-fold) was found in the liver at 12 h in the 1 × 10^9^ CFU/mL *S. agalactiae* treatment group (Figure 6A). In the spleen, its expression was slightly upregulated at 6 h only in the 1 × 10^9^ CFU/mL group, and it was significantly upregulated (*p* < 0.05) in a dose-dependent manner at 12 h (1.91 ± 0.15- and 4.67 ± 0.46-fold) (Figure 6B). In the head kidneys, gene expression levels were slightly induced at 6 h in both treated groups and only with 1 × 10^9^ CFU/mL treatment; however, the highest expression level was detected at 12 h (10.71 ± 0.52-fold) (Figure 6C).

**Figure 7:**
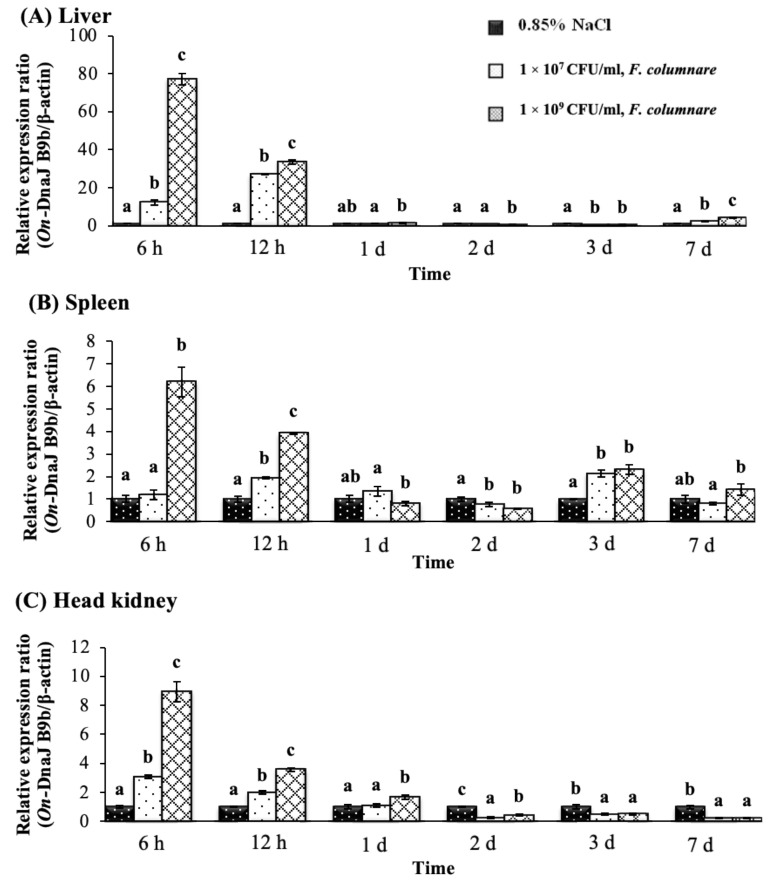
Nile tilapia *DnaJ B9b* transcript levels in fish exposed to *F. columnare* at 1 × 10^7^ and 1 × 10^9^ CFU/mL at different time points in the liver (**A**), spleen (**B**) and head kidneys (**C**).

In the *F. columnare* injection groups, *On-DnaJ B9b* transcriptional levels were found to be highly upregulated in a dose-dependent manner at 6 and 12 h (Figure 7A,C). In the liver, the highest *On-DnaJ B9b* gene expression level showed a 77.20 ± 3.03-fold induction at 6 h in the 1 × 10^9^ CFU/mL injection group (Figure 7A). In the spleen, only the 1 × 10^9^ CFU/mL treatment showed significantly upregulated expression of *On-DnaJ B9b* at 6 h, 12 h and 3 days, and the mRNA levels in all treated groups were dose-dependently induced (Figure 7B). In the head kidneys, transcript levels were significantly upregulated in a dose-dependent manner at 6 and 12 h. However, at 2–7 days, mRNA expression was downregulated at both concentrations (Figure 7C).

**Figure 8:**
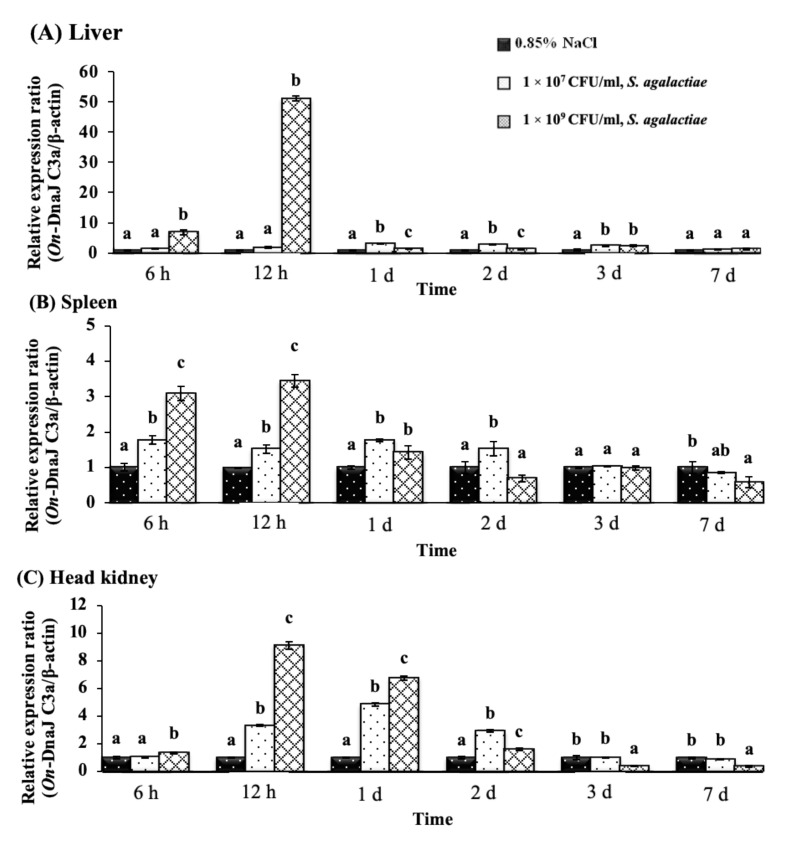
Nile tilapia *DnaJ C3a* transcript levels in fish exposed *to S. agalactiae* at 1 × 10^7^ and 1 × 10^9^ CFU/mL at different time points in the liver (**A**), spleen (**B**) and head kidneys (**C**).

The *On-DnaJ C3a* gene expression levels in fish after *S. agalactiae* injection with the 1 × 10^9^ CFU/mL treatment exhibited the greatest upregulation (51.09 ± 0.75-fold) in the liver at 12 h; however, the expression rapidly decreased to 1.60 ± 0.13-fold by day one (Figure 8A). In the spleen, this mRNA expression was slightly upregulated in a dose-dependent manner at 6 and 12 h, and the highest level was observed at 12 h (3.45 ± 0.18-fold induction) in the 1 × 10^9^ CFU/mL treatment group (Figure 8B). The *On-DnaJ C3a* gene showed prolonged expression in the head kidneys until two days after exposure, and a dose-dependent effect was observed at 12 h and 1 day (Figure 8C). The highest expression level (9.13 ± 0.27-fold) was found at 12 h in the 1 × 10^9^ CFU/mL treatment group.

**Figure 9:**
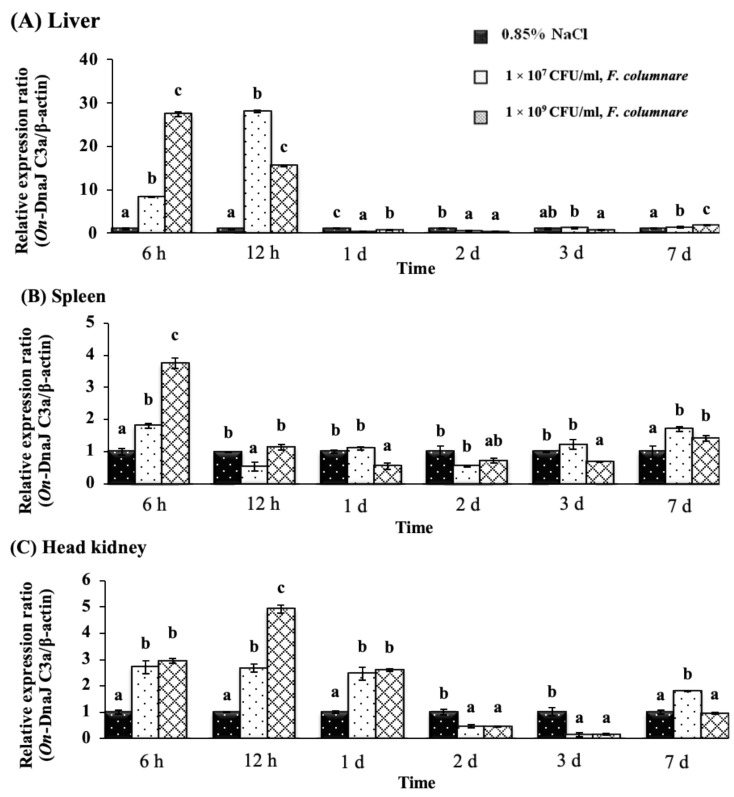
Nile tilapia *DnaJ C3a* transcript levels in fish exposed to *F. columnare* at 1 × 10^7^ and 1 × 10^9^ CFU/mL at different time points in the liver (**A**), spleen (**B**) and head kidneys (**C**).

In the *F. columnare* injection groups, *On-DnaJ C3a* mRNA in the liver was highly expressed at 6 and 12 h (Figure 9a). A dose-dependent expression pattern was only observed at 6 h, with the highest level (27.48 ± 0.54-fold) occurring in the 1 × 10^9^ CFU/mL treatment group; however, the expression later decreased to 15.51 ± 0.08-fold at 12 h (Figure 9A). In the spleen, a dose-dependent response was only found at 6 h, with the highest level (3.75 ± 0.17-fold) observed in the group of fish injected with 1 × 10^9^ CFU/mL (Figure 9B). In the head kidneys, the *On-DnaJ C3a* transcript level was increased in a dose-dependent manner from 6 h through one day (Figure 9C). At two and three days, *On-DnaJ C3a* gene levels were significantly downregulated (*p* < 0.05) in all treatment groups.

### 3.6. Gene Silencing Analysis

**Figure 10:**
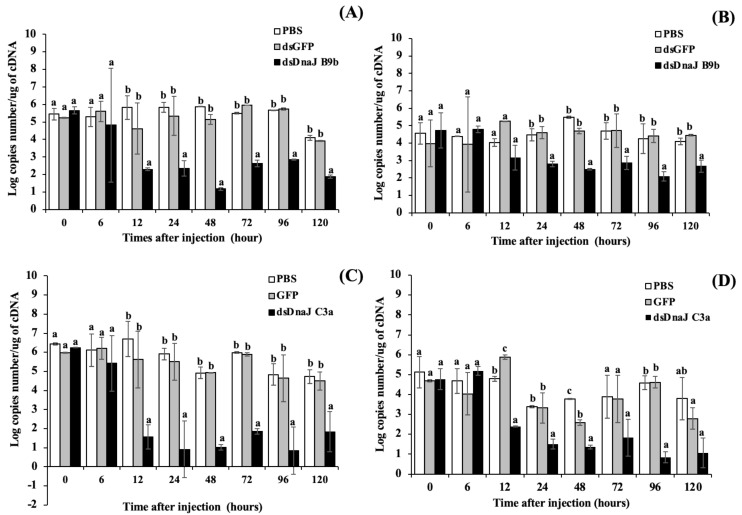
Silencing analysis in the gills (**A**,**C**) and liver (**B**,**D**) of the *On-DnaJ B9b* and *DnaJ C3a* genes, respectively, under normal conditions.

*On-DnaJ B9b* and *On-DnaJ C3a* expression was obviously knocked down in the target tissues (Figure 10A,C for the gills, Figure 10B,D for the liver). dsOn-DnaJ B9b and dsOn-DnaJ C3a strongly induced the degradation of the mRNAs of their target genes. At 12–24 h after treatment, these two dsRNAs effectively silenced the mRNA expression of the *On-DnaJ B9b* and *On-DnaJ C3a* genes. Significantly decreased mRNA levels of these two genes in the RNAi-treated groups were observed from 12 or 24 h through the end of the experiments at 120 h (*p* < 0.05) compared to those in the PBS- and dsGFP-injected groups.

### 3.7. Effects of Gene Knockdown under High-Temperature Stress and Coinjection with S. agalactiae

**Figure 11:**
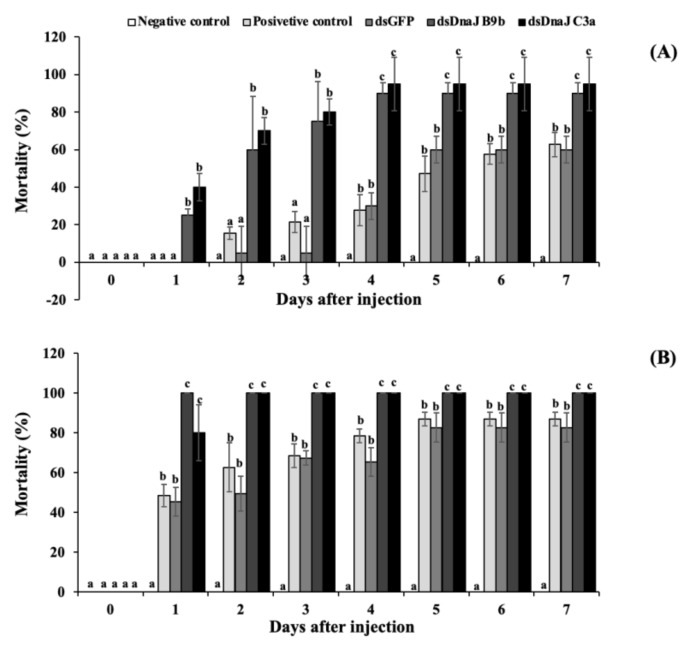
Effects of *On-DnaJ B9b* and *DnaJ C3a* expression knockdown on the mortality of Nile tilapia under high-temperature conditions (**A**). Negative and positive controls represent the PBS injection group at 28 ± 1.5 °C and the PBS injection group at 35 ± 2.1 °C, respectively. (**B**) High-temperature and *S. agalactiae* infection conditions; negative and positive controls represent the PBS injection group at 35 ± 2.1 °C and PBS-*S. agalactaie* injection group at 35 ± 2.1 °C.

Under high-temperature conditions, the fish injected with dsOn-DnaJ B9b and dsOn-DnaJ C3a initially showed rapid mortality of approximately 25.0% and 40.0%, respectively, and sunk to the bottom of the containers 1 day after injection. The highest mortality of these groups was observed at days four to seven, and especially at day seven, with mortality rates of 90.0% and 95.0%; these rates were significantly higher than those of the positive control group, dsGFP-injected group and negative control group, which exhibited mortality rates of 62.7%, 60.0% and 0.0%, respectively (Figure 11A).

High mortality was observed when fish were exposed to both high temperature and *S. agalactiae* infection (Figure 11B). In this part, the fish coinjected with dsOn-DnaJ B9b and dsOn-DnaJ C3a and *S. agalactiae* rapidly displayed high mortality at day one after injection, and some fish clearly exhibited erratic or lethargic swimming on the water surface. On day two, these two groups of fish exhibited 100% mortality with severe hemorrhage on pectoral fins and body surface, while in the positive control (PBS injection group in 35 ± 2.1 °C) and dsGFP-injected groups, which were coinjected with *S. agalactiae*, some of the fish showed clinical signs similar to previous groups with mortality rates of 62.7% and 49.5%, respectively. The mortality of these two groups gradually increased and stabilized at 86.8% and 82.7% during days five to seven. During this time, no mortality in the negative control group was observed until day 7 (Figure 11B).

## 4. Discussion

In this study, the full-length cDNA encoding the Nile tilapia *On-DnaJ B9b* gene was cloned and characterized. It was classified as a DnaJ protein type II subfamily member [23] because it is composed of a signal peptide, a J domain, and a glycine (G)/phenylalanine (F)-rich region [32]. The presence of a 22 aa leader sequence suggests that the On-DnaJ B9b protein needs to be translocated to the ER lumen to be further processed. This finding is supported by a previous report [27], in which ERdj4 was identified as a mobile soluble luminal protein found in the ER lumen. A glutamine insertion and a histidine-rich region (130–139 aa) are unique among vertebrates (Figure 1B), suggesting that On-DnaJ B9b may have acquired neofunctionalization. Furthermore, there was a lack of an N-linked glycosylation site (NKS) near the conserved HPD motifs; this site was very highly conserved among the DnaJ B9b molecules. However, On-DnaJ B9b does not have this carbohydrate-binding motif at this position or any other regions. This finding suggests that On-DnaJ B9b is not a glycoprotein, and it may have specific functions that are different from previously reported DnaJ B9a molecules from other species. Further study of its function is needed to clarify these important characteristics.

Molecular cloning and structural characterization of On-DnaJ C3a full-length cDNA in Nile tilapia were accomplished. Its cDNA is composed of a 24 aa signal peptide and one J domain, which revealed characteristics of type III Hsp40 [32] with ER-targeted proteins similar to those reported in a previous report [28]. It also has other characteristics of the HSP70 family [33] with multiple functions, such as protein-protein interactions involved in cell cycle regulation, transcription control, protein transport, neurogenesis and protein folding [34,35,36]. The function of DnaJ C3a is to maintain protein folding homeostasis during the UPR by reducing the misfolded protein burden and promoting protein refolding in the ER lumen via its TPR motif and accompanies BiP as a cochaperone [25,28,29].

3D structure analyses of both the On-DnaJ B9b and On-DnaJ C3a cDNAs revealed conserved HPD motifs in the J domain that are conserved in DnaJ protein families and are important for interactions with other partner molecules [23,33]. Both cDNAs contain 3’UTR instability motifs (ATTTA), and many instability motifs found in the 3’UTR cause the mRNA to be short-lived [37]. This finding suggests a short lifespan of *On-DnaJ B9b* and *On-DnaJ C3a* transcripts in microenvironments in response to ER stress, and these molecules may be rapidly degraded to save host bioenergy. A homology analysis between On-DnaJ B9b and On-DnaJ C3a (Supplemental material Tables S1 and S2) indicated that only the J domains were conserved between the cDNAs with relatively low amino acid identity and similarity. These data suggest that these DnaJ proteins have experienced evolutionary differences even though they are in the same family, as indicated by evolutionary tree analysis.

Finally, the evolutionary trees of both genes show high conservation among vertebrates, indicating similar fundamental functions in biological systems. These results suggested that DnaJ B9b (ERdj4) was more highly conserved in vertebrates than in invertebrates, and no homologs were found in *Caenorhabditis elegans*, yeast, or *Drosophila melanogaster* databases, as shown in a previous study [23].

A qRT-PCR analysis of *On-DnaJ B9b* gene expression in healthy Nile tilapia revealed high expression in the gonad and trunk kidney tissues. In the gonads, the new protein synthesis and/or misfolded protein overload rates may be higher than those in other tissues. Additionally, the weights of the experimental Nile tilapia used in this study were only approximately 30 g (premature stage); therefore, it is possible that the reproductive system in the gonad tissues is still continuously developing at this stage. Previously, it was found that *Mdg1* (*DnaJ B9b* or *ERdj4*) transcripts were more highly expressed in rat (*Rattus norvegicus*) testes than in other normal tissues and were significantly induced in rat mesangial cells that were exposed to methanol [22]. This finding suggests that the DnaJ B9b protein may play a vital role in translational regulation, especially in the gonads and kidneys. Additionally, *DnaJ B9b* (*ERdj4*) transcripts were highly expressed in the liver, placenta, and kidneys of humans [23]. Information from these studies suggested that these tissues contained well-developed intracellular ERs that produce many secreted proteins.

The highest expression of *On-DnaJ C3a* was observed in the gills, followed by the intestine, liver and trunk kidneys of normal Nile tilapia. It is likely that all these tissues have a high risk of exposure to numerous stressors, leading to increased misfolded/unfolded protein burdens that can overload cells, especially in the ER compartment [34]. Therefore, cells maintain cellular homeostasis by activating protein refolding, in which the On-DnaJ C3a protein and other ER chaperones contribute to a lower number of unfolded proteins. It was suggested that DnaJ C3a (P58^IPK^) is universally expressed in all tissues and plays a role in multiple stress responses, according to observations glucosuria, hyperglycemia and increased apoptosis in pancreatic islet cells from mutant mice with a P58^IPK^ deletion [33]. It was previously demonstrated that P58IPK−/− mice had excessive ER stress, increased apoptotic signals and exacerbated colitis. This finding suggests a critical function of DnaJ C3a in cytoprotection under cellular stress conditions [38].

In Nile tilapia, *F. columnare* and *S. agalactiae* were classified as severe pathogens that kill fish in slightly different manners. *F. columnare* can preferentially infect under conditions of rapidly fluctuating water quality, such as temperature and water hardness [39], while *S. agalactiae* is severely virulent at high water temperatures (>31 °C) [3,40]

We examined the expression patterns of the *On-DnaJ B9b* and *On-DnaJ C3a* genes in response to *S. agalactiae* and *F. columnare*, one of which is a Gram-positive bacterium and the other is a Gram-negative bacterium. *On-DnaJ B9b* transcript levels in the liver, spleen and head kidneys were significantly upregulated at 6 and 12 h and were slightly changed at one to seven days. These patterns were not different from those in different pathogenic infections, suggesting that fish can modulate misfolded/unfolded proteins by inducing the On-DnaJ B9b protein to activate misfolded protein degradation via the ERAD mechanism, which has been clearly demonstrated in mammalian cells [25,26,27]. Similar to the On-DnaJ B9b mRNA response, the *On-DnaJ C3a* transcripts in the liver, spleen and head kidneys were substantially induced in the early phase of infection within 1 day. This finding suggests that the fish body attempts to reduce misfolded protein burdens in the ER lumen by increasing On-DnaJ C3a protein levels. The basic function of On-DnaJ C3a in the UPR response has been well documented [25,28,38].

Acute phase proteins (APPs) are strongly produced in fish early following infection [41,42]. APPs are mainly synthesized in the fish liver upon the induction of cytokines and inflammatory mediators (IL-1, IL-6, and TNF-α) that are secreted into the plasma [41,42,43]. Based on this information, the liver is an important organ during the early phase following infection. Experimentally, the most substantial changes in *On-DnaJ B9b* and *On-DnaJ C3a* gene expression levels in the liver suggested that a fundamental function of hepatocytes is to inductively produce APPs with the help of various chaperone HSPs during synthesis and posttranslational processes. Furthermore, both pathogenic bacteria highly altered the expression levels of the *On-DnaJ B9b* and *On-DnaJ C3a* transcripts early after injection. These results suggested that these fish may use a variety of components in their innate immune responses to eliminate invasion during the early stage of infection. Additionally, in comparison among the tested organs, the livers of the infected fish showed strongly upregulated expression compared with the spleen and head kidney. This suggests that during an infectious state, the fish liver is the major organ maintaining bodily homeostasis by producing many APPs and other antimicrobial substances against bacterial invasion [41,42].

Recently, it was shown that hemolysin toxins, including streptolysin O (SLO) and streptolysin S (SLS), which are produced by group A *Streptococcus* (GAS), can induce host ER stress and UPR [44]. In our study, a group B Streptococcus (GBS) member *S. agalactiae* that induces β-hemolytic effects [45] could clearly cause major changes in *On-DnaJ B9b* and *On-DnaJ C3a* expression levels in the livers of the infected fish. These results suggest that GBS also induces ER stress and the UPR in fish. Additionally, *F. columnare* was found to highly upregulate the expression of these genes in the liver, suggesting that gram-negative bacteria have the potential to induce ER stress and the UPR in the host. It was also found that ER stress could be induced in lipopolysaccharide (LPS)-treated mice by increasing certain transcription factors (ATF4, X-box binding protein 1 [XBP1] and CCAAT-enhancer-binding protein [C/EBP] transcription factor [CHOP]) [46]. All of these factors are involved in cellular stress and apoptotic processes, which lead to acute lung injury in LPS-treated mice. This phenomenon suggests that these transcription factors, which are found in higher vertebrates following LPS-mediated induction, could also be involved in fish infected with Gram-negative *F. columnare*.

Generally, the host uses protein recognition receptors (PRRs) and APCs to respond to bacterial invasion via recognition of pathogen-associated molecular patterns (PAMPs) [47,48]. PAMP-PRR complexes can activate major intracellular signaling changes in host cells, resulting in a rapid induction in the expression of genes involved in inflammatory and immune responses [47,48]. These results suggested that Nile tilapia utilize PRRs to recognize *S. agalactiae* and *F. columnare* to trigger immunological responses. HSPs provide chaperoned polypeptides via MHC class II in APCs for specific triggering of the acquired immune response [48], suggesting that On-DnaJ B9b and On-DnaJ C3a are involved in antigenic processing in APCs during the infection period.

In fish, expression analyses of Hsp 40s have been intensively conducted in very few species. It was shown that the expression of 42 of 57 Hsp 40 in the gills, liver and intestine of channel catfish (*Ictalurus punctatus*) was strongly regulated in response to *Edwardseilla ictaluri* and *F. columnare*, especially in early infectious stages [49], suggesting their involvement in disease defenses in fish. The regulatory layer of these proteins, which governs the functional specificities of Dnaj cochaperones and their interactions with HSP70s, could be key to the wide range of cellular functions in the defense mechanisms of fish [50]. Upregulation of *Dnaj* expression after exposure to bacterial pathogens may strongly drive the interaction binding of heat shock factors (HSFs) and heat shock elements (HSEs) in HSP gene clusters to eventually produce the target heat shock proteins responding to those infectious diseases [51].

To date, information on the characterization and functional analyses of the *DnaJ B9b* and *DnaJ C3a* genes in fish is very limited. In the present study, functional analyses by RNAi techniques were first described in Nile tilapia. The dsOn-DnaJ B9b- and dsOn-DnaJ C3a-injected groups showed higher mortality (90–95%) than the control groups and induced very severe mortality of the tested fish (100%) within one to two days under *S. agalactiae* and heat coinduction. These results strongly demonstrate the crucial functional roles of these two proteins in both heat stress and pathogenic bacterial infection. The absence of these crucial proteins may seriously affect the UPR in the ER lumen [21]. Without ER DnaJs in UPR induction conditions, regulation and maintenance of the proper folding of impaired proteins in the lumen may not occur [23]. The lack of On-DnaJ B9b and On-DnaJ C3a might induce dysfunction of the ERAD pathway or prevent normal proteasomal degradation [25,26,27] or fail to maintain protein folding homeostasis and protein refolding in the ER lumen under UPR conditions [25,29]. These effects strongly induce susceptibility to both high-temperature stress and *S. agalactiae* infection in Nile tilapia.

## 5. Conclusions

This is the first report demonstrating the molecular cloning, gene organization and structural characterization of cDNAs encoding two novel ER chaperone genes in Nile tilapia. This information is crucially important for evolutionary research on DnaJ molecules. We also obtained valuable information regarding host-pathogen interactions by investigating host ER chaperone gene expression changes in both gram-positive and gram-negative bacterial invasion. The ER acts as a protein factory as well as a site for posttranscriptional modification and signals to other molecules that are involved in protein folding in response to ER stress; as shown in this study, the ER contains ER chaperones that mediate immune system responses to systemic pathogenic infections.

## Figures and Tables

**Table 1:** Oligonucleotide primers used.

Gene Name	Oligonucleotide Primer	Accession Number	Sequence (5’ to 3’)	Amplicon Size	Purpose of Use
*DnaJ subfamily B member 9b* (*DnaJB9b*)	*On*-DnaJ B9b F	KM_081674	GGAGACTACGACTTTAACCAGCAC	160	Real-time PCR, RACE
	*On*-DnaJ B9b R		CCTGGAAGTGGCTGTCAAAGTGTC	160	Real-time PCR, RACE
	DnaJB9b_F		GCCGAGAGATGCTACCGAGC	225	Gene knockdown
	DnaJB9b_R		GCCGAGAGATGCTACCGAGC		
	DnaJB9bT7_F		GGATCCTAATACGACTCACTATAGGGCCGAGAGATGCTACCGAGC	250	Gene knockdown
	DnaJB9bT7_R		GGATCCTAATACGACTCACTATAGGGCCCTCCCCTGATGAAGCAC		
*DnaJ subfamily C member 3* (*DnaJC3a*)	*On*-DnaJ C3a F	KM_081675	CAAGGATTATGTGACAGCTGCTGC	150	Real-time PCR, RACE
	*On*-DnaJ C3a R		TGGTGCTCAGCTTGTAGAAAGCCT	150	Real-time PCR, RACE
	DnaJC3a_F		CAGTGGCACCCGGACAACTT	225	Gene knockdown
	DnaJC3a_R		CAGTGGCACCCGGACAACTT		
	DnaJC3aT7_F		GGATCCTAATACGACTCACTATAGGCAGTGGCACCCGGACAACTT	250	Gene knockdown
	DnaJC3aT7_R		GGATCCTAATACGACTCACTATAGGCCCGGAGCCAAACGGATTGA		
*Green fluorescence gene*	GFP_F		TAATACGACTCACTAAGGGAGACACATGAAGCAGCACGACCT		Gene knockdown
(*GFP*)	GFP_R		TAATACGACTCACTATAGGGAGAAGTTCACCTTGATGCCGTTC		
*Beta-actin*	*On*-Beta-actin F	XM_003443127	ACAGGATGCAGAAGGAGATCACAG	155	Real-time PCR
	*On*-Beta-actin R		GTACTCCTGCTTGCTGATCCACAT	155	Real-time PCR

## Supplementary Materials

The following are available online at https://www.mdpi.com/article/10.3390/biom11101509/s1, Table S1. Homology analysis of the nucleotide and amino acid sequences of the *On-DnaJ B9b* gene. Table S2. Homology analysis of the nucleotide and amino acid sequences of the *On-DnaJ C3a* gene. Figure S1. Homology model of On-DnaJB9b. (A) Comparison with a nonredundant set of PDB structures of the On-DnaJB9b model and (B) local quality plot. Figure S2. Model of On-DnaJC3a homology. (A) Comparison with a nonredundant set of PDB structures of the On-DnaJC3a model and (B) local quality plot. Figure S3. Phylogenetic trees of the Nile tilapia *DnaJ B9b* and *DnaJ C3a* genes.
