# Supplementary material for: Molecular Characterization and Defense Functions of the Nile Tilapia (Oreochromis niloticus) DnaJ B9b and DnaJ C3a Genes in Response to Pathogenic Bacteria under High-Temperature Stress Conditions
Source: Biomolecules. 2021 Oct 13;11(10):1509. doi: 10.3390/biom11101509 (PMC8533496; doi:10.3390/biom11101509)

Supplemental materials Table S1.

DnaJ B9b	Accession number	Nucleotide identity (%)	Amino acid	
			Identity (%)	Similarity (%)
**Mammals**				
Human	NM012328	64.6	62.4	80.2
Cattle	NM001193039	63.2	61.6	81.1
Mouse	NM013760	63.9	62.3	80.2
Rat	NM012699	63.7	62.3	79.7
**Avian**				
Chicken	NM001030735	63.0	60.9	77.1
**Amphibian**				
Frog	NM001087324	60.9	64.7	79.3
**Teleost fish**				
Salmon	BT046349	30.4	25.6	33
Zebrafish	NM001025184	66.6	62.9	81.1
Pufferfish	XM003972823	77.7	77.7	90.3
Japanese medaka	XM004082887	77.3	80.9	91.2

Srisapoome et al. (2021)

Supplemental materials Table S2.

DnaJ C3a	Accession number	Nucleotide identity (%)	Amino acid	
			Identity (%)	Similarity (%)
**Mammals**				
Human	NM006260	66.8	68.8	84.5
Cattle	BC126580	67.2	68.7	84.7
Mouse	NM008929	66.0	68.1	84.1
Rat	NM022232	66.5	67.5	82.7
**Avian**				
Chicken	NM001008437	67.3	68.5	84.3
**Amphibian**				
Frog	NM001086630	68.0	69.8	85.9
**Teleost fish**				
Salmon	NM001140557	77.5	81.7	90.7
Zebrafish	BC049406	76.3	81.0	92.5
Pufferfish	XM003961822	80.9	85.3	93.8
Japanese medaka	XM004081777	85.2	88.5	95.0

Srisapoome et al. (2021)

Supplemental materials Figure S1.

Homology model of On-DnaJB9b. (A) Comparison with non-redundant set of PDB structures of the On-DnaJB9b model and (B) local quality plot.

A

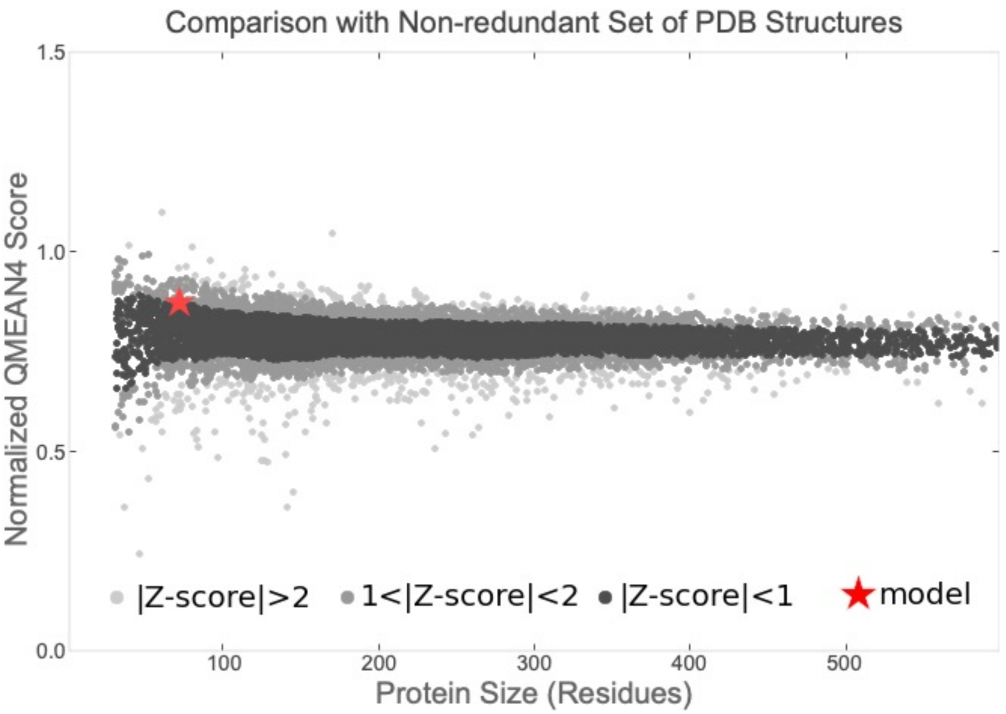

B

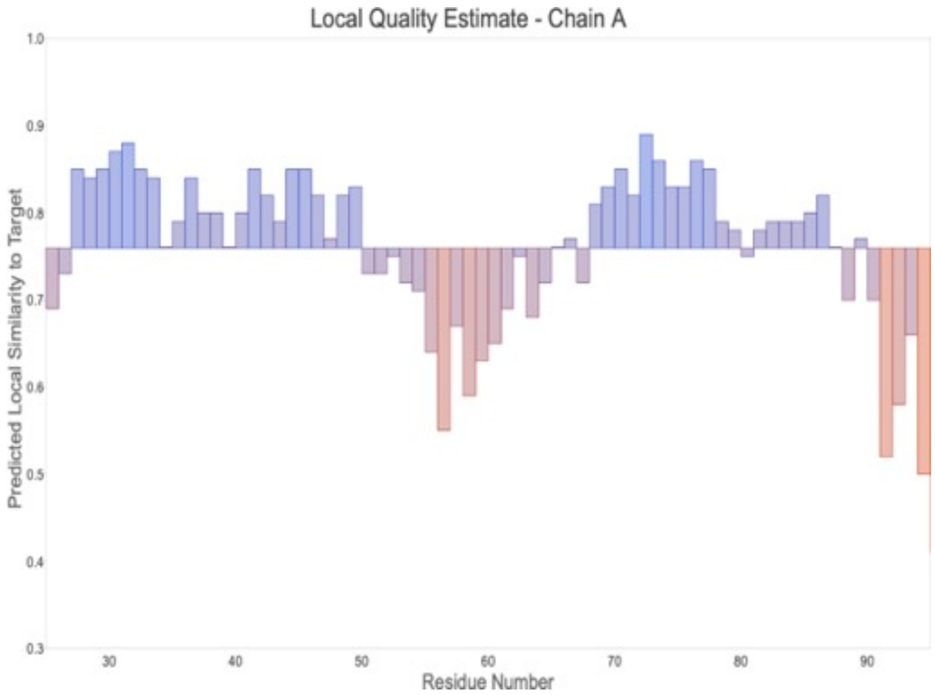

Supplemental materials Figure S2.

Homology model of On-DnaJC3a. (A) Comparison with non-redundant set of PDB structures of the On-DnaJC3a model and (B) local quality plot.

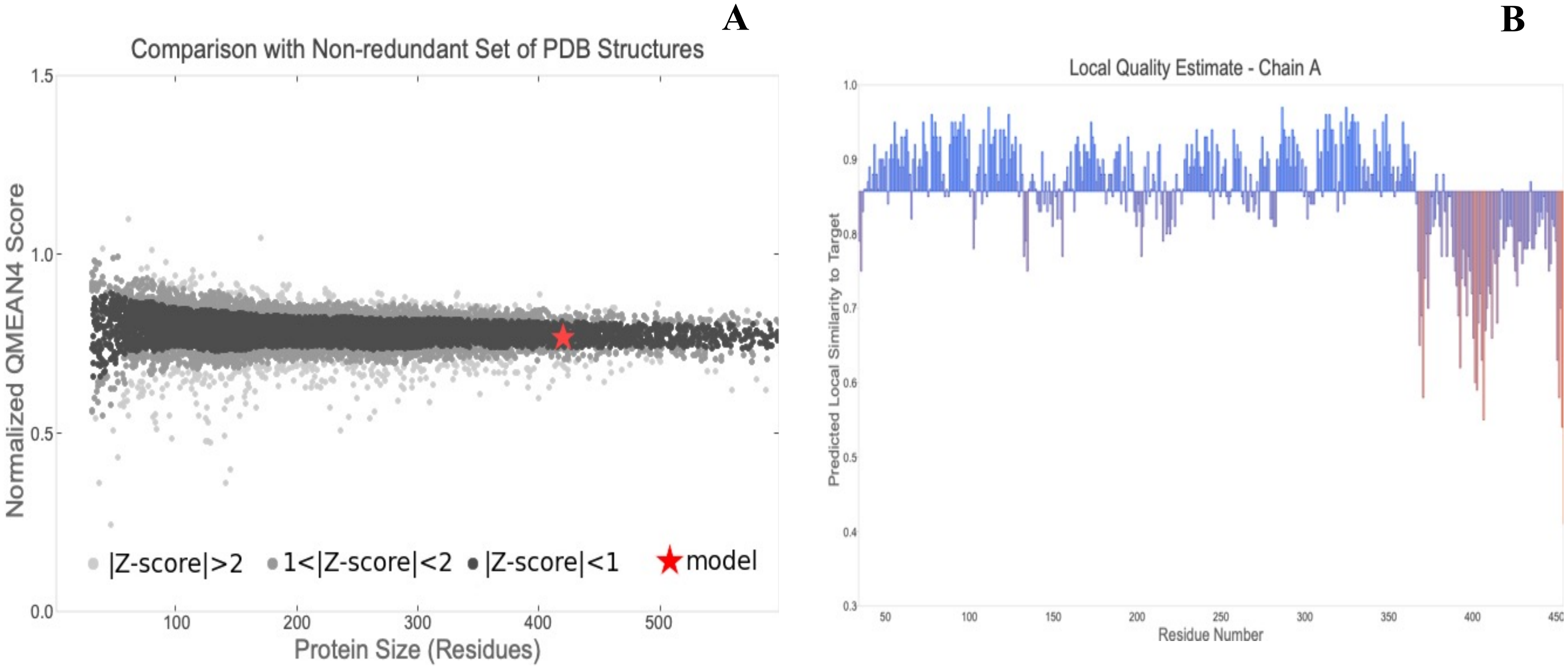

Supplemental materials Figure S3.

Phylogenetic trees of the Nile tilapia *DnaJ B9b* and *DnaJ C3a* genes. The trees were constructed with the UPGMA method using 1,000 bootstraps. The scientific and common names for each organism are labeled in each branch of the *DnaJ B9b* and *DnaJ C3a* genes. The GenBank accession numbers of the *DnaJ B9b* and *DnaJ C3a* genes of each species are indicated in parentheses.

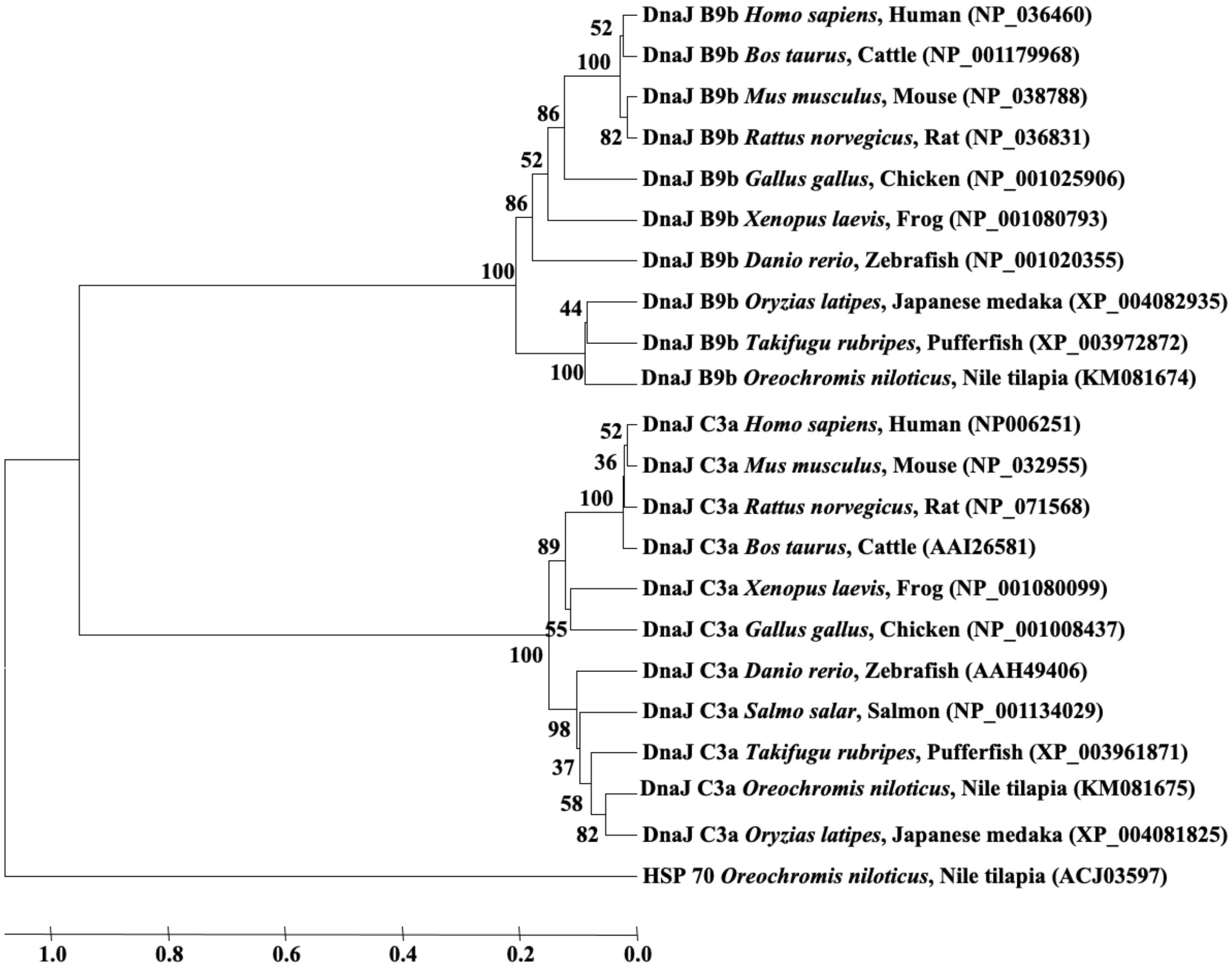

Supplement: Supplementary file 1 [file biomolecules-11-01509-s001.zip › biomolecules-1374251-supplementary.pdf]
